# What’s in Your Heart? Development of a Culturally Grounded and Trauma-Informed Parenting Support Program with a Pacific Northwest Tribe

**DOI:** 10.3390/ijerph22081253

**Published:** 2025-08-10

**Authors:** Sara F. Waters, Meenakshi Richardson, Alvina Marris, Fawn Harris, Myra Parker

**Affiliations:** 1 Department of Human Development, Washington State University Vancouver, 14204 NE Salmon Creek Avenue, Vancouver, WA 98686, USA; fawn.harris@wsu.edu; 2 Prevention Science Program, Washington State University Vancouver, 14204 NE Salmon Creek Avenue, Vancouver, WA 98686, USA; mrich106@jh.edu; 3 Center for Indigenous Health, Department of International Health, Johns Hopkins University, 415 N Washington Street, 4th Floor, Baltimore, MD 21231, USA; 4 Behavioral Healthcare Program, Coulee Medical Center, Grand Coulee, WA 99133, USA; amarris15@gmail.com; 5 Department of Psychiatry and Behavioral Sciences, University of Washington, 1959 NE Pacific Street, Box 356560, Seattle, WA 98195, USA; myrap@uw.edu

**Keywords:** Indigenous, program development, culturally grounded, trauma-informed, intervention, parenting

## Abstract

The aims of the current study included gathering cultural knowledge and stories regarding parenting young children within a Tribal community and learning community members’ perspectives on key components of a promising parenting prevention program. Qualitative data were drawn from a focus group including seven participants and semi-structured phone interviews with 21 additional participants, all of whom were parenting children in the community. Hybrid coding and applied thematic analysis revealed five themes: (1) Desire to Learn and Gain Parenting Skills; (2) Relationships and the Caregiver Role; (3) Culture and Caregiving, which included subthemes of Diversity Among Tribal Bands, Intergenerational Knowledge Sharing, and Reconnection and Revitalization; (4) Historical Trauma and Behavioral Health; and (5) Curriculum Terminology Considerations. The information gathered collectively informed the development of the culturally grounded *stim’ aspuʔús* (*What’s in Your Heart?*) parenting program. This addresses the great need for culturally grounded interventions to support trauma healing within Indigenous families. The development process and implications for program development by and for Indigenous communities is discussed.

## 1. Introduction

The Indigenous populations in the U.S. experience inequitable rates of poor mental, physical, and behavioral health outcomes, which are rooted in experiences of historical and contemporary trauma exposures caused by colonization and ongoing settler-colonialism. These traumas impact not only individuals, but move through families, are transmitted intergenerationally, and permeate communities. Healing from these traumas, then, involves individuals nested within their familial and community relationships. For Indigenous peoples, cultural connectedness is integral to the pathways towards healing and greater wellbeing. Despite the evidence for cultural connection as a protective factor for Indigenous peoples [1], few trauma-informed prevention programs for Indigenous families are grounded in cultural knowledge and practices [2]. In the current paper, we share the development of a culturally grounded, trauma-informed parenting program with and for a Pacific Northwest Tribal community. This project involved a community-academic research partnership and centered around community leadership and Indigenous worldviews to address the needs of Tribal families.

### 1.1. Colonization and Historical Trauma

For the Indigenous peoples of North America, known to many Tribes as Turtle Island, colonization has included experiences of genocide, dispossession of land, forced removal and relocation, spiritual oppression, and cultural disconnection [3,4,5,6]. Most salient to the context of Indigenous families is the government-mandated program of residential or boarding schools throughout the U.S. and Canada, which was designed specifically to disrupt Indigenous systems of care by forcibly removing children from their families and communities [7]. These practices continued through the Indian Adoption project, 1958–1967, in the U.S. and the “Sixties Scoop” in Canada, in which Indigenous children were systematically targeted by the respective child welfare systems, removed from their families, and placed with (white) foster families [8]. Today’s dramatically inequitable rates of child welfare involvement among Indigenous families are the extension of these efforts [9].

The experiences of colonization have produced historical trauma, which refers to the “cumulative emotional and psychological wounding over the lifespan and across generations, emanating from massive group trauma experiences” [10], within many Indigenous communities. Behaviors associated with the historical trauma response include depression, anxiety, anger, difficulty coping, and maladaptive emotion regulation [10]. Historical trauma manifested as intergenerational trauma adversely impacts family systems and healthy child development within many Indigenous communities [3,5], putting children at risk for the profound biobehavioral effects early trauma exposure has on lifespan development [11]. There is a great need for interventions to support healing from trauma among Indigenous families and that need has largely gone unmet.

### 1.2. Culturally Specific Trauma Interventions

While there is growing recognition of the need for trauma-informed care within many systems and communities, there is still a marked lack of trauma-informed interventions for Indigenous peoples [12,13] and for Indigenous caregivers, who may be parents, grandparents, relatives, or other community members, and children, in particular [2]. There are some notable exceptions, including an intergenerational program for First Nations and Metis female caregivers and their daughters [14] as well as the Boomerangs parenting program for Aboriginal parents and young children [15]. Nevertheless, most behavioral health programs and interventions are based on a Eurocentric worldview and developed by and for dominant Western populations. Such programs have often failed to serve Indigenous peoples effectively [16] and cultural specificity may be critical for strengthening impact. One meta-analysis revealed that, among 76 culturally adapted mental health interventions with over 25,000 participants, the culturally adapted interventions were more effective than the unadapted versions and that interventions that targeted a specific cultural group were the most effective by far [17]. For Indigenous peoples, participation in cultural and traditional practices and other forms of cultural connectedness are key sources of resilience [1], suggesting that effective interventions for Indigenous peoples must involve these cultural components.

There are multiple paths by which to adapt a program or intervention, bringing it into greater cultural alignment with a specific group for whom it was not originally developed [18]. Program adaptation can be represented on a continuum from non-adaptation through surface structure adaptation to deep structure adaptation [19]. Surface structure adaptation might involve swapping original program imagery out for images with greater cultural relevance, while deep structure adaptation might involve swapping original program content for content with greater cultural relevance. Situated at the far end of the continuum is culturally grounded interventions, in which the cultural context, knowledge, and practices of the specific community are at the center of the program. Culturally grounded interventions may include components drawn from the evidence base of Western science, but these pieces are integrated into the intervention through a deeply collaborative process that prioritizes the role of community voice throughout [19]. The *stim’ aspuʔús* (*What’s in Your Heart?*) program shared in the current study is one example of a trauma-informed, culturally grounded intervention.

### 1.3. Guiding Frameworks

The community-based participatory research (CBPR) approach is most appropriate for developing a culturally grounded intervention with an Indigenous community because it recognizes and advances the knowledge and value systems of a given community to improve the health and well-being of that community [20]. CBPR principles center cultural perspectives and community involvement [21], are committed to equity and power-sharing among partners [22] and foster an open dialogue to build relationships with community members and ensure that cultural considerations and community concerns are shared [23]. Communication is “the driver of disrupting power hierarchies… central to empowerment, both in seeking shared power for community members within partnerships and in disrupting social and health inequities” [24] (p. 365) While the use of CBPR principles is increasing, it remains under-utilized within Western applied science disciplines. Implementation of CBPR principles can ensure that the community informs and invests in the culturally grounded programs aimed to support them [25].

A CBPR approach aligns with the key theoretical frameworks underpinning the current work, namely Indigenous Ways of Knowing (IWK) and the Indigenous Connectedness Framework (ICF). As a theory, IWK refers to worldviews rooted in respect, relationality, and reciprocity [26]. Research informed by IWK centers Indigenous knowledge, prioritizes sharing of stories, and uplifts both individual and collective voices [27]. IWK has supported the growth of strength-based ‘culture as prevention’ approaches to behavioral health interventions within Indigenous communities. Introduced by an Inupiaq scholar, the ICF identifies the key factors informing the healthy development and wellbeing of Indigenous children [28]. Drawing from the extant research literature and knowledge shared by Elders and other community members regarding conceptualizations of child development, the framework places child wellbeing at the intersection of five mechanisms of connectedness: Family, Community, Intergenerational, Spiritual, and Land/Place. The ICF is also foundational to the model of Indigenous relational wellbeing in early childhood [29]. In this model, early relational wellbeing is at the center surrounded by Spirituality, Culture, and Ceremony followed by Relational Practices of the family, community, and intergenerational relationships. Our work presented in this paper is guided and informed by these approaches and frameworks.

### 1.4. The Partnership and Current Study

A Tribal-academic research partnership established through CBPR was formed in 2016 in response to the commitment of the Tribe’s Native American Research Center for Health (NARCH) to provide caregiving programming to support trauma healing among tribal community families. Tribal leaders and researchers identified the need for a trauma-informed caregiving support program in their community and expressed interest in the promising universal preventive intervention Attachment Vitamins (AV) [30] because of its trauma-informed lens, focus on child development, group format, and leadership by trained facilitators who are community members and do not need to have advanced degrees. The AV parent group program involves 10 weekly meetings with topics and exercises aimed to increase caregivers’ (1) knowledge of how toxic stress and trauma affect children’s development and behavior; (2) attunement to the emotional needs of the child; (3) mindful awareness of “moments of connection” with the child; (4) executive functioning skills including emotion regulation; and (5) ability to reflect on themselves and their parenting [31]. The curriculum introduces the concept of angels in the nursery as a counterpoint to ghosts in the nursery [32] and differentiates between “angel” moments, when the caregiver recalls childhood memories of moments when they felt especially loved, understood, or protected, and “stormy” moments, when the caregiver recalls childhood memories of moments when they felt unloved or unsafe. In 2017, the third author was trained in the AV parent group program and facilitated a group with parenting members of the Tribal community.

While Tribal leadership and members of the research partnership found value in some aspects of the AV parent group program, they understood that traditional caregiving knowledge, beliefs, and practices held by the community had to be at the center of the program. Thus, cultural adaptation was needed. The Tribal-academic research partnership team conducted a qualitative study with the primary aim of identifying community members’ perspectives on cultural components of parenting/caregiving young children to center these knowledges, beliefs, and practices related to parenting in development of the culturally grounded *stim’ aspuʔús* (*What’s in Your Heart?)* program curriculum. The secondary aim was to determine the acceptability of specific components of the AV program such as “angel” and “stormy” moments through community members’ reactions to these terms to inform the extent to which these aspects of the AV program would be incorporated into the *stim’ aspuʔús* (*What’s in Your Heart?)* program.

## 2. Materials and Methods

### 2.1. Author Positionality

Consistent with Indigenous methodologies and an ongoing commitment to self-reflexivity [33], we acknowledge our identities and knowledges as they relate to the current research. The first author is a white woman, mother, daughter, sister, and auntie living in the Pacific Northwest. Her research sits at the intersection of developmental and prevention science. She has been partnering with the NW Tribal community to support culturally grounded research to heal intergenerational trauma for nine years. The second author is a citizen of the Haliwa-Saponi Tribe and of Indo-Fijian descent residing in the Pacific Northwest. She has collaborated with both reservation-based and urban Indigenous communities for ten years to support public health promotion and prevention strategies that are culturally informed and community based. The third author is a member of the Colville Confederated Tribes. She has worked as a clinical psychologist serving Tribal communities for over 15 years. She is motivated by her family and community to improve mental healthcare and access to mental health for Tribal communities. She is also strong advocate for incorporating Indigenous ways of knowing into mental healthcare. The fourth author is a white woman with formal training in psychology, human development, and social work. She lives in the Pacific Northwest and brings over twenty years of experience in parenting. Her clinical work is grounded in a strengths-based framework that emphasizes clients’ existing capacities and resilience, and she holds an endorsement from the Washington State Association for Infant Mental Health for Culturally Sensitive, Relationship-Focused Practice. For the past five years she has worked collaboratively with the Northwest Tribal community and has done so with cultural humility as she recognized the expertise of each person through their unique journey. The fifth and last author is Mandan and Hidatsa and is an enrolled member of the Three Affiliated Tribes in North Dakota. She is also Cree on her father’s side, from the Rocky Boy Indian Reservation in Montana. She has a Ph.D. in public health, with a focus on health services along with a J.D. and M.P.H. She has worked with the Tribal partner community as a researcher and an evaluator on several research projects and evaluation efforts since 2011. Her research work includes cultural adaptation of evidence-based interventions, Indigenous research ethics, community-based participatory research, and incorporating Indigenous knowledge approaches into research and evaluation.

### 2.2. Participants

Qualitative data were gathered from a purposive sample of 28 community members (75% female) who identified as parents or caregivers of children 10 years or younger, elders or language experts, or as early childhood education professionals. Inclusion criteria also included being an English speaker, 18 years or older, and residents of the NW Tribal community. Participants were recruited through outreach to the Tribes’ Language program and early childhood education program, an interest sign-up sheet at a Tribal membership meeting, and word-of-mouth or snowball recruitment.

### 2.3. Procedures

It was confirmed alongside community partners that data through story would be gathered via a multi-day process wherein research team members gathered in person with participants in focus groups planned for March 2020. However, only one focus group, which included seven members of the Tribes’ Language program, could be held before data collection was disrupted by the COVID-19 pandemic shutdown. The focus group protocol was then revised to a one-on-one, semi-structured interview conducted by phone. Twenty-one participants completed phone interviews with a member of the research team. The protocol for the focus group and subsequent phone interview, developed in consultation with community partners, included open-ended prompts regarding cultural perspectives on parenting/caregiving (e.g., Please describe how you view culture as related to parents’ roles) aligned with our primary aim as well as open-ended questions eliciting feedback regarding specific terminology from the original AV curriculum. The concepts of Heart Moments, Angels in the Nursery, Angel, and Stormy Moments were explored to address our secondary aim. Participants did not have direct experience with the AV curriculum, but were introduced to key program concepts and terminology during the focus group or interview (e.g., Now that you are familiar with the definition of “Stormy Moments,” what is your opinion of this term? Can you provide a culturally specific term that you would prefer?). Please see Supplementary Materials for interview questions. Approval for study protocols were obtained through the Tribal review process and university institutional review board processes prior to data collection.

Code saturation is, in data analysis, the point at which no new codes are identified and meaning saturation the point at which no new nuances or insights are identified. Our sample is well aligned, based on prior research [34] to identify the range of topics surfaced (i.e., code saturation) as well as establishing a deeper understanding of those topics (i.e., meaning saturation). The focus group and interviews were audio-recorded and transcribed for data analysis.

### 2.4. Data Analysis

A codebook of parent (primary) and child (secondary) codes was developed by the first, third, and fifth authors via hybrid coding, an approach that uses both inductive and deductive coding strategies [35]. All authors reviewed and coded a subset of transcripts independently, then convened for code conferencing to refine the codebook and coding protocol. This process allowed for author accountability in discussing cultural relevance and the role of author identity and self- reflexivity, both Indigenous and White allied, in data analysis [26]. Then two pairs of authors (i.e., first and third author, second and fourth author) applied codes to transcripts using Atlas.ti, and inter-coder reliability testing was conducted to achieve a Krippendorff’s Alpha of 0.8, or an 80% reliability rate. There were no discrepancies between coders. Upon hybrid coding, applied thematic analysis was employed by the second and fourth authors [36], involving a systematic review of transcripts to group codes to identify descriptive themes that surfaced across the dataset.

## 3. Results

Hybrid coding yielded four parent codes: (1) Relationships and Roles, (2) Parenting Knowledge, (3) Course Processes; (4) Course Concepts, the first two corresponding to our primary aim and the second two corresponding to our secondary aim. A total of 15 respective child codes were identified. Applied thematic analysis surfaced four themes that addressed our aim of gathering information about cultural perspectives on parenting/caregiving: (1) Desire to Learn and Gain Parenting Skills, (2) Relationships and the Caregiver Role, (3) Culture and Caregiving, and (4) Historical Trauma and Behavioral Health. The fifth theme, Curriculum Terminology Considerations, addressed our secondary aim of learning community members’ impressions of key components of the AV curriculum. Exemplar quotes were selected and presented with respect to cultural significance throughout the following subsections expanding upon themes and corresponding subthemes.

### 3.1. Desire to Learn and Gain Parenting Skills 

The theme Desire to Learn and Gain Parenting Skills is related to cultural revitalization and intergenerational relationships to ensure the transfer of knowledge and cultural parenting practices that are not predominantly carried out in practice. Participants shared their beliefs that many parents in the community are lacking skills due to several factors, specifically related to not having knowledge passed down by their own parents and young parents caregiving without support or education, seeking “how to” guidance. Participants acknowledged two-fold perceptions of intergenerational relationships and the effect on parenting skills. The first being position, recognizing how family relationship carry a lot of weight, and trauma passed between generations were voiced as negative. One respondent stated,

I think the community needs more parenting classes. We have a lot of young moms that, sometimes they didn’t get raised with mom and dad. It’s a lot of community raising community. So when these young women are having babies, they don’t really know what they’re doing because they were raised by their aunties, cousins.

In addition, while there is a perceived need for additional parenting skills among community members, it was noted that if parents feel they may be judged, even those looking for the “how to’s” may find it difficult to fully engage. One respondent stated,

The parenting class is an excellent idea. I just think that some people think of it as a bad thing. Like saying, “Oh well, they’re saying I don’t know how to parent…trying to educate parents a little bit more of what it actually is. Like the information says this is what this type of class is.

Respondents provided insight into how although there is great interest for parents to gain cultural parenting knowledge, many noted not having been raised in a traditional sense. Participants identified that there are not resources consistently accessible or available. One participant indicated that:

So we weren’t raised culturally, like traditionally, but I do have some aspects of that, of being around my grandparents up here. So I know about what to do during a funeral and stuff like that, but not fully cultural. And I feel now that I’m an adult and have children of my own, I wish I did know that because culture is a lot for our members and we just don’t have the resources. Like, we do, and we don’t.

### 3.2. Relationships and the Caregiver Role

Participants spoke to the connection between that of children and community members. From a traditional perspective, many spoke to how the disciplining of children by other community members was an accepted norm. Although, acceptability depends on the parent(s) and the given situation. Notably, one participant shared

It takes a village and if my child is in the wrong and is doing wrong, I expect and encourage my family and people in my community to hold them accountable. They understand and a lot of people that my children are raised around know and understand what I expect of them. So, I would appreciate it if they held them accountable and whatnot.

Generally, it was shared that most continue to accept the norm of community members stepping in to offer support and guide children, and there is an understanding that boundaries are set and not to be disrespected, nor the parental role dismissed in comparison to other caregivers. Participants noted that it is acceptable for adults in the community to “discipline” children or report back to parents as long as it’s not in a harmful way. Participants emphasized that caring for and guiding children is viewed as the communities’ responsibility to collectively look out for youth in caring, compassionate and respectful ways. Several participants shared their opinion that it is more acceptable for someone other than a parent to be involved in discipline if there is an established relationship with the child. One individual shared:

I guess it depends on who it is and what the situation is. Most of the time in this kind of community, it’s to look out for one another’s children. And also to step in when it is needed if there’s not, you know, a parent present or an adult present and a child is in danger. It’s part of the community’s responsibility to step in and do the right thing.

Participants shared that intervention by Elders in the community would be more acceptable. The expansive support that children traditionally have from extended family and community members is a strength. Others shared how although spread out geographically, families are still very connected, and these connections lend support to the younger generation. One respondent noted,

It’s kind of like a ripple effect where you meet somebody and that person introduces you to somebody else and somebody else and that’s kind of how I think of it. Once you fit in with a family member and get ties, they always welcome you in their home or in their knit unit.

Participants expressed that many children lack traditional experiences directly from their parents and community relationships enrich children’s experiences. For example, one woman discussed how each summer while her family was working, community members would provide her and her brother with comfort and security, sharing that,

I remember as a little, tiny girl that my aunties and, my mom and dad’s siblings, uncles and stuff, would do a lot of comforting for me, not necessarily my parents, but because we all had extended families back then. I think comfort comes from many different adults and [older] children.

### 3.3. Culture and Caregiving

Participants described how the role of culture in caregiving is integral for engaging, meeting and interacting with others within the community reinforced by traditional teachings and the resurgence and continuation of traditional caregiving approaches:

Culture is related to our role in pretty much everything that we do. We do a lot of things based on our traditions and the values and beliefs that we’ve been taught and learned through our own lives.

Amongst respondents, there was a general reference to situations and experiences being dependent on how the individual, family, parent, or caregiver was raised themselves. And moreover, how those experiences have influenced behaviors, interactions, and perceptions surrounding how they themselves care for children:

Even though I was raised off the reservation, we were [taught] to hunt, fish, and camp, and visit relatives on the reservation. And even though I wasn’t raised around my language and…with people culturally, even though they brought it to our house…because of the [Elders]…that used to come, I think there was an understanding of how to raise and how to do things culturally, even though it wasn’t firsthand all the time.

Participants also emphasized the role of other caregivers and cultural influence, transitions and identity formation in raising and teaching their children. Another shared:

Keeping my kids in tuned with a lot of the cultures…and values that I was raised with by my…parent, my mom, and my grandparents is what I try to instill in my children, because it’s part of who we are as a people. I think it’s important to keep those cultures alive by teaching my kids.

The theme of Culture and Caregiving is further delineated into the following subthemes: Diversity among Tribal Bands, Intergenerational Knowledge Sharing, and Reconnection and Revitalization.

#### 3.3.1. Diversity Among Tribal Bands

Throughout the interviews participants were asked to provide information about their views and what they have been exposed to in terms of cultural differences between Tribal bands. Mainly, participants shared about differing traditions, languages and practices between bands, and addressing the importance in respecting and acknowledging the heterogeneity within a given Tribal community or reservation. One participant shared that it was imperative that the community continues to stay mindful that Tribes within their reservation are indeed different.

Within the Tribe [there are] actually bands of Tribes, so those bands, we all practice our culture differently and even with the Tribes that are sister Tribes of us, we all practice and speak differently, so keeping that in mind and being respectful on if you are attending a cultural practice of another band or another Tribe to either ask permission if it’s okay that you practice the way you practice or if they would prefer if you practice the way they do.

The protocols rooted in respect surrounding, recognizing, and practicing traditions of other Tribal bands was highlighted in an effort to express the diverse needs of community members within the reservation. Additionally, one participant shared that there are different ceremonies, protocols, and expectations of how children should act and behave in those settings. Specifically, it was noted that between the different bands that there are “very distinct, strict protocols of ceremony and where children are allowed and how they’re expected to act”.

Moreover, participants shared responses that surfaced themes relating to the cultural differences between Tribal and non-Tribal practices. Specifically, regarding differing traditions and parenting practices and those who were raised and live on reservation vs. off. There was consensus that any differences are mainly attributed to the way one was raised. One participant shared that:

It would depend on where you were raised. I know a lot of our non-members were raised partially Tribal because of their friends, that a lot of our non-members have the same upbringing as Tribal members do. And some even have more cultural aspects of their parenting plan than I would just because I wasn’t raised fully cultural, traditionally, like some of my family members were.

Participants also referenced similarities between bands, such as powwows, horse racing, smoke houses and winter dances as practices that are found across bands, although the ceremonies and teachings surrounding those practices differ band to band and even between areas of the reservation (e.g., districts).

#### 3.3.2. Intergenerational Knowledge Sharing

The value of ceremony, tradition and relationship building is integrated into the upbringing of future generations. In terms of intergenerational knowledge sharing between that of Elders and younger generations, an interviewee shared that:

I think as long as the Elders are respected than they treat the younger people with respect, and kind of show them that it’s okay. And guide them once the other gets respect, and then they know the child will listen or wants to learn, in addition ideals that are raised to respect others… and we have a lot of Elders that try to pass on teachings. And so, I think that does trickle down to the younger generations.

In response to cultural perspectives on behaviors and temperaments, participants shared vivid oral tradition stories, Tribal language references, and teachings specific to emotional responses (i.e., crying). There was a general agreement among participants surrounding the benefits and vitality of intergenerational parenting, caregiving, and disciplining with the inclusion of Elders, parents, and community members. One participant shared, that depending on the caregiver, the child will express different behaviors and temperaments, stating that:

I guess you can see different temperaments and children depending on which families they come from and whether they’re being raised by their parents or maybe like an Elder or something like that. A lot of the children that are being raised by Elders, have a lot [of] different temperament compared to others.

Participants described that parental roles and caregiver-child relationships are defined more so through the lens of emotional connection rather than a family with hierarchical, or nuclear family systems. One participant made a reference to multi-generational homes as being common, by stating:

I see you being raised by Elders more than their parents living under the older generation, I kind of feel like they have more of an insight to their tradition in a way. And so that’s important to them and they seem that they can listen and behave a little better.

Some participants noted that although they themselves were not raised culturally or traditionally—although some were raised by their grandparents; they see much value and importance in fostering and accessing resources to elevate cultural integration and experiences for their children. Participants expressed the need for more opportunities to learn about culture and traditions, especially as it pertains to caregiving roles, encouraging that others would immerse themselves in it to pass that along to their children and the next generations. Specific to language, one participant stated:

I would put my whole heart into our culture ‘cause I wasn’t raised [that way], and I cannot speak Salish and it sucks because I can’t teach my children that.

#### 3.3.3. Reconnection and Revitalization

There was an overarching call for returning to and nurturing the continuation of cultural protocols, such as introducing oneself to one another within the community and storytelling from a cultural perspective:

If it’s an Elder, you let them know who you are, who your parents are, and who your grandparents are. That way, you can potentially find that long lost relative you’ve never known because somehow we are all related on the reservation just because we are all part of different areas of the State, but they migrated us to the current Reservation. So, to introduce yourself culturally, they want to know your family.

This quote addresses historical forced removal and highlights the reconnection to the community by engaging in introductions and interactions, such as sharing who their family is, which in turn, reignites the connection between relatives and by extension knowledge sharing throughout the reservation. Another respondent echoed the previous quote stating:

It’s the polite thing to do to say your name, and where you’re from, and who your family is, your parents and your grandparents. And that sort of gives people an opportunity to see how they’re tied together, or if they’re tied together, um, through family.

Participants emphasize children being connected with Elders and IWK, that enriches values and reactionary behaviors. One participant also shared that it has been difficult to reignite cultural traditions and pass them on generation to generation: “we seem to have lost a lot of that. I come from a real lost generation myself that is…we’re making a conscious effort to return to some of that village to raise a child thing”. A common throughline across all interviews was to address these barriers by reconnecting to people, place, and tradition to foster stronger relationships with others and with the land. 

Participants expressed the cultural importance of revitalizing storytelling and oral traditions, as they are used to teach lessons and instill values early on in a child’s life. Traditionally, stories are passed down and are used to answer “why” questions, and to outline expectations for behavior and relational dynamics:

It would be through a story or…show you something and tell you, ‘This is why you don’t do it.’ It [is] pretty experiential. It’s like an experience, you actually see it.

One respondent also shared the importance of when stories are told as well as the importance of accuracy and delivery of storytelling in practice,

That’s one thing about storytelling, our storytelling, we’re supposed to remember exactly the way it’s told to us and repeat it that way... that isn’t always easy, but some families have their own stories. They can pass on and pass down. And then when you get mixed with other families then you can share those. And we have a thing about storytelling that it’s like being a couch potato. We don’t tell stories during our gathering seasons when we’re gathering, when we’re berry-picking, when we’re hunting...Our storytelling is when we’re shut in during the winter months and repairing and mending and making, whether it’s new clothing, new tools, renewing our tools or whatever it is, like fish nets or whatever, that’s when we’re allowed to do storytelling. And there are stories that go along with why we set our food traditionally, the first food that gets set is like the moose, or whatever that meat is. And then the salmon and then the roots, because that’s the subsistence cycle that we’re being thankful for and honoring. And that’s why we have the peace.

### 3.4. Historical Trauma and Behavioral Health

The impact of historical trauma on parenting practices emerged through interviews with participants speaking to the negative implications of boarding schools and oppression intergenerationally and its impact on the parental role, accessing, and utilizing behavioral health resources. With regards to boarding schools, a participant shared:

I see that, to some degree, a lot of us, either our parents or grandparents were boarding school generation. And so, there’s some really lack of parenting skills that’s been passed on. And, I don’t really know how to say it. I’m not saying that non-Tribal people make better parents ‘cause that would not be true, but a lot of those parenting skills got lost and are slowly being regained.

The generational trauma linked to boarding schools found in family dynamics was shared and how it has and continues to impact participants directly. A parent shared that: “like myself, my mother was a boarding school kid, so she really didn’t know how to be a mom; that’s just how it is”. Moreover, the spiritual oppression brought forth by boarding schools also differs between families, role dynamics, and Tribal bands. A participant explained that:

I was raised Catholic, but I also was raised not religiously. I went to the boarding school here, but my mom didn’t forcefully make us [practice Catholicism]. And that’s sometimes how I feel with [Tribal band], that you’re forced to do it this way whether you like it or not. And then on my side…my grandma told me, “If you don’t like it, don’t do it. I don’t care.” But if I was [Tribal band], I wouldn’t have a say so.

The insight into the connection between that of one’s culture, caregiving role, and responsibility speaks heavily to the need to specify, define and share the role of a parent and the caregiver-child relationship considering the dynamic contexts of culture—they cannot be separated, as they are intertwined and woven together to shape the wellbeing of the community and families.

Participants described that historical trauma, discrimination, and ongoing oppression has severely influenced behavioral health access and care. General perspectives of behavioral health services were shared, many of whom expressed barriers to service utilization, limited resources, financial strain, a lack of trust of providers and direct services available, and concerns about confidentiality. There was an overarching commonality amongst the participants, in addressing that these barriers are not new, but rather have been exacerbated into the present day. There were also concerns discussed regarding practitioners on the reservation being non-Tribal members, predominantly White, and often condescending toward parents or caregivers. The lack of cultural humility and education throughout receipt or processes contributes to apprehension in accessing support. A participant shared that:

If we talk to a non-member and we call them, we will talk to a non-member but in our statement we will say White people. They take offense to that. And I don’t think being in that profession they should take offense to it because that’s how, culturally, we feel. Because we grew up with a White people stigma and how they treat us poorly. We need more behavioral health professionals on our reservation.

Overall, there was an overarching call for elevated and culturally centered behavioral health programming and education. As one participant mentioned, “the better healthcare, better mental healthcare we can have and everything, better the cycle will get on the reservation”. In turn, that focus, and positive transformation will break trauma cycles and uplift generations to come.

### 3.5. Curriculum Terminology Considerations

Participants were asked to share feedback on course concepts as outlined in the AV curriculum, specifically with regards to language references and appropriate considerations for cultural adaption. Course concepts are outlined as subthemes: Heart Moments, Angels in the Nursery, Angel Moments, and Story Moments.

#### 3.5.1. Heart Moments

The course concept of Heart Moments is defined as those moments of connection between parent or caregiver and child. Examples of these bonds were outlined as common practice. Among participants, there was an overarching response relating to nurturing and providing comfort for children, with an emphasis on the connection and relationship building between caregiver and child. With one parent saying “hold them close and please take your time and pay attention”. Additionally, “if your child is crying or upset or whatnot then basically just nurturing them” is considered a Heart Moment. One participant expressed their awareness of these moments and shared their experience of not having had those bonds and the importance of uplifting those connections:

I was raised around non-Indians whose dads used to pet their hair and look into their eyes and say, “You’re so beautiful. I just love you. You’re just cherished.” Who does that? You know? And where’d he come from? What family is this? You know, that isn’t my family.

#### 3.5.2. Angels in the Nursery

Responses varied based on reaction to the term *Angels in the Nursery*, as some provided personal experiences in reaction to the term once heard. Thoughts of protection and being watched over were the most commonly noted response. When given the definition and asked their opinion, participants thought the term related to someone or something protecting a child. A participant shared that:

I definitely believe that it’s a factual, true thing…simply because in my own experience, I told my daughter stories while she was in the womb and when she was born and she was crying, as soon as I started speaking she calmed down. So, I believe that- that nurturing thing, even in the womb, it’s a beginning of a good relationship between a parent and a child.

For some the hearing of the term brought up feeling of ancestral connection: “I guess if I were to think about it, angels and the nursery to me, it would be like an ancestry thing ‘cause angels make me think of like ancestry and something like that and so”. In addition, interpretations of peace surfaced, as one participant indicated that “I guess that’s the perception, that an angel is like peace or tranquility”.

When asked if there may be a different term to use as opposed to Angels in the Nursery, participants predominantly provided a general hesitant reaction with the overall use of the term, associating angels with death. A participant provided insight into integrating or editing the term within a generational context:

We pray for the next seven generations. This is something that I tell my kids, that seven generations ago, they prayed for us. And seven generations from now, we’ve got to pray for them. And we fought for survival then and so we have to do it now for the sake of seven generations ahead. And so that just puts some value on our ancestors and our future.

This response heavily relates to the request to relate terminology used throughout the curriculum to align with, relationality, emotional response, intergenerational, and ancestral bonds. Another participant offered an alternative to the term in reference to nurturing and connections to the natural world, suggesting the use of an animal as opposed to an ‘angel’, “like [as] nature-based concept, you know?... So, like [an] animal.” There was also a general opposing view of the term with its reference to Christianity and Catholicism, and their mission, conversion and religious overcast:

To me that, angels in the nursery sounds more like a Catholic Christian type deal. If it came to my children and my teachings. But I’m not saying I’m against it. Because I believe in free will and I express this to my children.

Another participant shared, “I probably wouldn’t use those specific terms, in large part because of the deity behind it.” Most respondents were against the use of the term, but did not provide a cultural term in its stead, although one individual stated that: “I know a lot of Natives, myself included, don’t necessarily believe in God, more so of a Creator. So I think that would probably be a conflict, using the term angels”.

#### 3.5.3. Angel Moments

Responses varied based on reaction to the term, at times tied to a personal experience. Participants noted that the term Angel Moments, as described alongside its definition regarding positive or healthy interactions between a caregiver and child. Most respondents found the term acceptable based on the context and definition. Although, one respondent provided an alternative approach to the term:

That [is a] Grandma character, you know, that’s imprinted at a young age, or that special auntie, whoever the one that wiped your tears and made you feel better and gave you a cookie. Not that there’s anything wrong with the term angels, but we have stories about grandmothers doing good things. And we don’t have stories about angels.

Participants’ thoughts provided insight into other cultural references that could be used in parallel to the terms’ definition. Grandparents were identified as being central to relationship-building, family systems, and traditional storytelling, suggesting references to grandparents and Elders, as opposed to ‘angel’, since the term is not found within traditional and cultural contexts. Participants also expressed their apprehensive feelings toward the use of the term ‘angel’ as it again relates to Christian, Catholic, and religious tones. One participant shared that, “just the angel part just sounds religious.” Another shared that, “even if people did have that connection to God and stuff, the angel portion, most people feel like a passing-on type of thing. That just seems weird to me”.

In a like manner, other participants noted that although they understood what was meant by an Angel Moment, they believed using the word “angel” may be too literal for some caregivers, especially those looking for a more culturally traditional, less Western experience. Some participants acknowledged that simply removing the word “angel” and using “happy moments” or “good moments”, would seem more culturally relevant. One respondent answered with:

I would say loving moments, you know. Like, what are your happy memories? What are your happy thoughts? Type of deal.

#### 3.5.4. Stormy Moments

In relation to the Stormy Moments, referring to moments of difficulty in bonding or transitions in emotion regulation (e.g., tantrums), responses varied based on reaction to the term, but were mainly positive. Participants shared personal experiences and views in relation to the definition of Stormy Moments. One parent shared that the term was particularly relevant to changes in emotions, noting that, “our life is a series of storms and calms and usually the storm changes things and the calm heals things, so that term is right on point”. Another respondent shared that the term was relevant to responding to difficult moments when children may be “they’re throwing fits and that they need to take a moment to sit down and relax and let the storm pass”. Most respondents confirmed finding the term acceptable based on the context and definition, although one respondent felt that the term did not sit well with them, as they felt it may bring about negative feelings or responses to unsafe experiences:

I think the unsafe and the unloved is probably touchy. Maybe there might be a different way to describe that just because of our connection to the generational trauma, so that might be something to reconsider using those terms.

## 4. Discussion

Trauma-informed interventions that are tailored to specific cultural contexts are needed to support the wellbeing of Indigenous families. In this paper, we shared the results of a qualitative study conducted to inform the development of just such a culturally grounded parenting intervention, designed to support families in a NW Tribal community on their journey of healing from intergenerational trauma. Our primary aim to gather perspectives and understandings from community members regarding the cultural knowledge, beliefs, and practices of parenting young children was addressed by identification of the first four themes that emerged.

The first theme, which spoke to the desire expressed by participants for greater access to cultural parenting practices, affirmed the value held by the community for a culturally grounded parenting program and reaffirmed the importance of the research team’s larger objective. One of the primary strategies of the colonization of Turtle Island has been disruption of Indigenous family systems and interruption of the intergenerational continuity of cultural knowledge [7]. This theme acknowledges this fact while elevating the resilience of Indigenous families and revitalization of culture.

The second and third themes spoke to the integral role of culture in all aspects of caregiving young children. The importance of ceremony and Tribal protocols and the significance of learning these practices through relationships with elders, the land, and the broader community were emphasized. The value of community caregiving or having extended family members and community members actively involved in parenting and raising young children was highlighted. The focus on kin and community members as caregivers challenges the dominant model in Western child development research, which elevates the primacy of the dyadic child-caregiver bond [37]. Thus, an emphasis on a broadened conceptualization of who are parents/caregivers in Indigenous communities supports decoloniality, particularly within Indigenous early childhood mental health and child welfare [38,39]. The second and third themes also fit well with the connectedness mechanisms articulated in the ICF including Spiritual, Intergenerational, and Community connectedness [28].

Particularly noteworthy is the fact that many participants identified the need for behavioral health services, represented in the fourth theme, within the community and explicitly articulated the value of a parenting intervention to support healing from trauma. It was generally expressed that this parenting intervention should be in service to the call for cultural revitalization and reconnection. In fact, some participants discussed their desire to raise their children with greater cultural connectedness and communicated barriers that stood in the way of achieving this goal. Thus, the need for a culturally grounded parenting program, which was identified by Tribal leadership, was echoed consistently by caregiving community members. The complexity of developing a culturally grounded program was underlined as well though, as there are multiple distinct bands in this Tribal community and the need to recognize and honor differences among these groups was brought forth.

The secondary aim of the qualitative study was to ascertain parenting community members’ perspectives on the terms specific to the AV parent program. As represented in the fifth theme, moments of connection between caregiver and child, a central component of the AV program, were discussed in terms of Heart Moments, echoing the title *What’s in Your Heart?* The importance of connection, nurturing, and comfort or safety within the child-caregiver relationship came through for participants with this term. The phrase Angels in the Nursery, which was developed as a strengths-based alternative to the concept of ghosts in the nursery [30], did not resonate for many participants. A few even offered more culturally relevant conceptual alternatives, such as imagery linked to ancestral ties, spirituality, or connection to more-than-human kin (e.g., animals, plants). Similarly, the term Angel Moments was not well aligned culturally and evoked unwanted associations with Catholic and Christian religiosity for some. In contrast, responses to the term Stormy Moments were largely positive, highlighting its relevance and the power of its imagery, even if the subject itself is difficult.

### 4.1. Curriculum Development

Having achieved both the primary and secondary aims of the qualitative study, the Tribal-academic research team used the information gathered from the study to develop the *stim’ aspuʔús* (*What’s in Your Heart?*) program for small groups of community members who are parenting/caregiving a young child, whether they are parents, grandparents, aunties, uncles, or other kin, in alignment with the second theme which highlighted the importance of relationships. Each section of the curriculum was developed to include facilitated discussion of some trauma-informed information about child development, as indicated by the fourth theme of the association between historical trauma and mental health. This content was woven with cultural practices and storytelling, in alignment with the third theme of culture and caregiving. Some of the concepts and terms from the AV curriculum were reconstructed and incorporated into the *stim’ aspuʔús* (*What’s in Your Heart?*) program, as indicated by the fifth theme. Guided by the first and third themes in particular, the research team worked with language and cultural experts in the community to develop a set of six land-based cultural family activities that include traditional language words and brief guidance on cultural protocols. Information about a different activity would be provided to participants in the program following each session, with different sets of activities tailored to each season. The entire curriculum was shared with members of the Tribal Language Program over a series of in person meetings and further refinement to the materials was made based on their experiences and feedback to finalize the *stim’ aspuʔús* (*What’s in Your Heart?*) program curriculum.

### 4.2. Considerations

There are considerations and learnings from this qualitative study and the resulting curriculum development process that may be relevant for other Indigenous communities with similar programs or interest in developing them. Firstly, we elevate the critical importance of centering Tribal sovereignty in this work. This meant that the need for a trauma-informed parenting program was determined and articulated by the Tribal community, not by the academic partners or other external agents, that leaders of the community were integral members of the research team, and that community members directly informed and shaped all aspects of the research study and the program curriculum that emerged from it. Related to this was a foundational commitment of the research team to privileging community strengths, particularly cultural traditions, as a source of resilience and to the critical praxis of relationship-building, walking alongside, and intersectional cultural humility [40]. For those pursuing similar culturally grounded program development, we offer our learnings regarding the need for understanding the impacts of colonization unique to and within each Indigenous community as well as the need to remain sensitive to differences in awareness of historical trauma and knowledge of cultural practices among community members. We elevate the deep investment of time required for this kind of work and the thoughtfulness and intentionality this effort requires for long-term sustainability of the program and partnerships.

### 4.3. Limitations

Limitations of this study include the limited generalizability of the findings. The work reported in this paper includes the voices and perspectives of members of one NW Tribal community. While there are cultural commonalities among Tribal communities, there is great diversity among the more than 500 federally recognized Tribes and many more unique bands. We do not presume that the information brought forth from our participants is necessarily applicable across these communities. Such generalizability was not the primary goal of this study. Another limitation may be the one-on-one phone interview format of much of the data collection. Responding to the semi-structured interview questions in the focus group format, as was originally planned, may have led to quantitatively or qualitatively different information as participants would have had the opportunity to elaborate or build upon one another’s perspectives or provide contrasting points of view. Nevertheless, pivoting to the phone interview format allowed the work to continue during a difficult and uncertain time amidst the COVID-19 pandemic.

## 5. Conclusions

In this manuscript, we shared caregiving community members’ stories and perspectives regarding the child development and the child-caregiver relationship from a CBPR study. This information shaped the development of a culturally grounded parenting program to support healing of intergenerational trauma within a NW Tribal community. This project was initiated by Tribal leadership based on the community-identified need for such a program and the work was conducted in partnership among academic researchers and key community members. The content, including supporting material like cultural activity handouts, of the *stim’ aspuʔús* (*What’s in Your Heart?*) parenting program was reviewed thoroughly by Tribal cultural and language experts before the curriculum was finalized. The result is a parenting support program by, of, and for this specific Tribal community, centering cultural connection and revitalization within family systems as the pathway to healing. We hope the sharing of this work will support other Tribal communities to develop culturally grounded parenting programs of their own.

## Data Availability

The raw data supporting the conclusions of this article will be made available by the authors on request.

## Supplementary Materials

The following supporting information can be downloaded at: https://www.mdpi.com/article/10.3390/ijerph22081253/s1, File S1: Focus Group/Interview Questions.

## Abbreviations

The following abbreviations are used in this manuscript:

CBPR	Community Based Participatory Research
IWK	Indigenous Ways of Knowing
ICF	Indigenous Connectedness Framework
AV	Attachment Vitamins
NW	Northwest
