# Supplementary material for: What’s in Your Heart? Development of a Culturally Grounded and Trauma-Informed Parenting Support Program with a Pacific Northwest Tribe
Source: Int J Environ Res Public Health. 2025 Aug 10;22(8):1253. doi: 10.3390/ijerph22081253 (PMC12386269; doi:10.3390/ijerph22081253)
Supplement: Supplementary file 1 [file ijerph-22-01253-s001.zip › ijerph-3760764-supplementary.pdf]

Focus Group / Interview Questions

Please describe how you view culture as related to parents’ roles.

Are there different parenting practices between the bands of the Tribe? If so, please describe some examples.

Are there different parenting practices between Tribal members and non-Indians? If so, please describe some examples.

Do community members reinforce certain behaviors/rules/values/customs for children between the ages of 0 and 10 years of age? If so, please describe some examples.

What are some traditional teachings or stories about infants that you know of?

What are some stories about ways children learned how to behave?

What are some stories or teachings about how to deal with tantrums?

Now I’m going to read a definition of “angels in the nursery” – what is your opinion of this term? Can you provide a culturally specific term that you would prefer?

Now I’m going to read a definition of “stormy moments” – what is your opinion of this term? Can you provide a culturally specific term that you would prefer?

Now I’m going to read a definition of “angel moments” – what is your opinion of this term? Can you provide a culturally specific term that you would prefer?

How do community members perceive support programming or help seeking for developmental issues?
